# Adherence to exercise referral schemes by participants – what do providers and commissioners need to know? A systematic review of barriers and facilitators

**DOI:** 10.1186/s12889-016-2882-7

**Published:** 2016-03-05

**Authors:** Fiona Morgan, Alysia Battersby, Alison L Weightman, Lydia Searchfield, Ruth Turley, Helen Morgan, James Jagroo, Simon Ellis

**Affiliations:** Specialist Unit for Review Evidence (SURE), Cardiff University,1st Floor, Neuadd Meirionnydd, Heath Park Campus, Cardiff, CF14 4YS UK; National Institute for Health and Care Excellence, Level 1A, City Tower, Piccadilly Plaza, Manchester, M1 4BD UK

**Keywords:** Exercise Referral Scheme, Primary care, Physical activity promotion, Commissioning

## Abstract

**Background:**

Physical inactivity levels are rising worldwide with major implications for the health of the population and the prevalence of non-communicable diseases. Exercise referral schemes (ERS) continue to be a popular intervention utilised by healthcare practitioners to increase physical activity. We undertook a systematic review of views studies in order to inform guidance from the UK National Institute of Health and Care Excellence (NICE) on exercise referral schemes to promote physical activity. This paper reports on the participant views identified, to inform those seeking to refine schemes to increase attendance and adherence.

**Methods:**

Fifteen databases and a wide range of websites and grey literature sources were searched systematically for publications from 1995 to June 2013. In addition, a range of supplementary methods including, a call for evidence by NICE, contacting authors, reference list checking and citation tracking were utilised to identify additional research.

Studies were included where they detailed schemes for adults aged 19 years or older who were ‘inactive’ (i.e. they are not currently meeting UK physical activity guidelines). Study selection was conducted independently in duplicate. Quality assessment was undertaken by one reviewer and checked by a second, with 20 % of papers being considered independently in duplicate. Papers were coded in qualitative data analysis software Atlas.ti. This review was reported in accordance with PRISMA (Preferred Reporting Items for Systematic Reviews and Meta-Analyses statement).

**Results:**

Evidence from 33 UK-relevant studies identified that support from providers, other attendees and family was an important facilitator of adherence and ‘making exercise a habit’ post programme, as was the variety and personalised nature of sessions offered. Barriers to attendance included the inconvenient timing of sessions, their cost and location. An intimidating gym atmosphere, a dislike of the music and TV and a lack of confidence in operating gym equipment were frequently reported.

**Conclusions:**

These findings provide valuable insights that commissioners and providers should consider. The main themes were consistent across a large number of studies and further research should concentrate on programmes that reflect these findings.

## Background

Physical inactivity levels are rising with major implications for the prevalence of non-communicable diseases and the general health of the population. According to the World Health Organisation, “physical inactivity is now identified as the fourth leading risk factor for global mortality” [1]. Revised UK Chief Medical Officers’ guidelines for physical activity recognise that this activity can help prevent and manage over 20 conditions and diseases including coronary heart disease, some cancers, diabetes, obesity and musculoskeletal disorders [2]. Current recommendations suggest a minimum of 150 min of moderate or 75 min of vigorous exercise weekly combined at least twice weekly with activities to increase muscle strength and reduce sedentary behaviour [2]. In children, physical activity levels decrease from the age of 11 onward with a greater reduction for females than for males [3]. Recent data from the UK indicates that whilst around two thirds of those aged 16 and older met physical activity guidelines, this declines significantly with age [4]. A tool examining the Health Impact of Physical Inactivity indicates that, across England, only 21 % of people aged 40–79 achieve the recommended minimum weekly exercise target and major health gains could be made if this percentage was increased [5]. This lack of physical activity could cause as many as 36,815 premature deaths in England annually, signalling a compelling need for interventions that reduce inactivity.

A wide range of approaches have been explored to increase physical activity. Examples include population level interventions such as changes to the enviroment. Others operate at an individual level, such as brief advice from primary care practitioners; considered a key setting for the promotion of physical activity [6]. A larger number of primary care-based interventions have been developed over the past 20 years [7]. Exercise referral schemes (ERS), first established in the early 1990s [8], consist of an assessment involving a primary care or allied health professional to determine that someone is inactive, a referral to a physical activity specialist or service, an assessment to determine what programme of physical activity to recommend and participation in that programme [9].

Exercise referral schemes continue to be popular despite a lack of evidence for their overall effectiveness and cost-effectiveness [10–12] in reducing inactivity. In 2006, the National Institute for Health and Care Excellence (NICE) issued guidance entitled “Four commonly used methods to increase physical activity” which included ERS. That guidance stated there was insufficient evidence to make any decision on ERS efficacy and recommended schemes only be commissioned as part of properly designed and controlled research studies [11]. In 2014 guidance specifically on exercise referral schemes was issued [9]. It states that funding should be restricted to sedentary or inactive individuals with existing health conditions or other factors putting them at increased risk of ill health. Schemes should also incorporate core techniques outlined in separate guidance on behaviour change (NICE public health guidance 49) [13].

To inform the deliberations of the NICE Public Health Advisory Committee considering exercise referral schemes [9], we undertook a systematic review of views studies [14]. The review sought to determine the factors influencing referral to, attendance at and successful completion of exercise schemes and longer-term participation in physical activity from the perspectives of those using, and those providing, commissioning and delivering these services. In the full review, clear themes emerged from the views of participants that, if taken into account, could maximise chances of ERS success and are therefore relevant to all who commission and provide such programmes. This paper reports on participant views with the aim of informing those seeking to refine schemes to increase attendance and adherence.

## Methods

This review is reported in accordance with the PRISMA (Preferred Reporting Items for Systematic Reviews and Meta-Analyses) statement [15].

A protocol was agreed with NICE. Following this, fifteen databases and a wide range of websites and grey literature sources were searched systematically to identify relevant studies in the English language conducted between 1995 and June 2013. A range of supplementary methods including a call for evidence by NICE, contacting authors, reference checking and citation tracking were utilised to identify further research.

Studies were included where they detailed views of ‘inactive’ adults aged 19 and older referred to exercise referral schemes (i.e. those not currently meeting UK physical activity guidelines). Where the age range was below 19 years, studies were included if most participants were aged 19 or older. When individuals were referred to exercise schemes for health reasons other than rehabilitation, they were assumed to be inactive. Schemes had to include assessment and referral by a primary care or allied health professional; formal assessment by a physical activity specialist; a physical activity programme.

Study selection was conducted independently in duplicate. All studies were assessed using the appropriate NICE quality appraisal form [16]. No quality appraisal form was available for process evaluations which were therefore not appraised. Quality appraisal [16] and data extraction were undertaken by one reviewer and checked by a second, with 20 % of papers considered independently in duplicate. Papers were coded in qualitative data analysis software Atlas.ti [17] by one reviewer and were checked by a second.

A thematic analysis of the evidence was completed guided by NICE and Dixon Woods et al. [16, 18]. Views from cross-sectional and mixed methods studies were analysed thematically and integrated with key findings from qualitative studies.

## Results

**Fig. 1:**
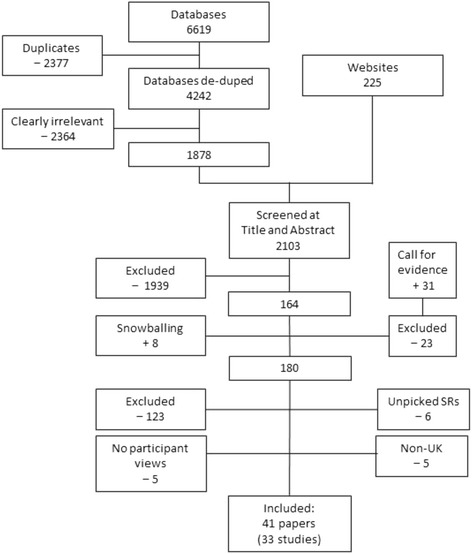
Flow Diagram

From database and website searching 6844 citations were identified, of which 180 papers were reviewed in full text. Given the size of the evidence base from UK studies, non-UK studies were excluded unless they contained data for hard to reach populations such as ethnic minorities, people with disabilities, and those experiencing socio-economic disadvantage. Forty-six papers describing 34 UK studies and one non-UK study met the inclusion criteria. Of these, 41 papers from 33 studies had data on participant views [See Fig. 1].

Twenty-four qualitative studies, eight cross-sectional and one longitudinal were included. Of the qualitative studies, two [19, 20] were assessed as high quality [++], fourteen [21–34] as moderate quality [+] and eight [35–42] as low quality [−]. Six [43–48] of the eight cross-sectional studies were deemed moderate [+] and two [49, 50] low quality [−]. The one prospective longitudinal study [51] was assessed as high quality [++].

Thirty-two studies were UK-based and one [28] was conducted in the Netherlands. The studies are summarised in Table 1 and details are available in the full review [14]. Six studies [20, 22, 26, 27, 32, 40] were reported in two papers each and one study [24] in three papers.

**Table 1:** Summary of included studies

First Author & year	Design & Quality	Intervention	Venue	Duration (weeks)	Population
**Beaufort Research 2013** [49]	CSS^c^	Welsh National ERS	LC	16	Wales; N = 1000; Age ≥18
**Beers 2006** [21]	Qualitative^b^	Free access to advice and facilities – ELC participants	LC	12	Wirral; N = 181; Age 16–79
**Carroll 2002** [19]	Qualitative^a^	GP referred - activities including gym, swimming and aerobics	LC	Varied	Mid and North England; N = 35; South Asian Muslim women; plus 10 GP referrers, 13 scheme providers
**Clarke 1996** [43]	CSS^b^	GP referred ERS with personalised counselling and tailored exercise prescription	LC	12	Birmingham; N = 500; mean age ca 46 [SD ca 14.0]; 69 % F; 40 % social class IV/V
**Cock 2006** [35]	Qualitative^c^	GP referred ERS (5 schemes); activities included gym-, water- and hall-based exercises	LC	10–13	South & North England; N = 1024
**Crone 2002** [22]	Qualitative^b^	GP referred ERS (3 schemes)	Varied	Ca 12	North & South West England; N = 18; mean age 55.5 years; F13 M5
Crone 2005 [54]					
**Cummings 2010** [44]	CSS^b^	Exercise for Health programme; walking, gym, swim, cycle and class-based exercises	Not stated	Not stated	Northern Ireland; N = 210; mean age 54.8 ± 15.7; F106 M104
**Day 2001** [50]	CSS^c^	Exercise for Health programme; consultation & follow up	Varied	8	Scottish borders; N = 324
**Graham 2006** [23]	Qualitative^b^	GP referred ERS - Consultation, exercise, review.	LC/G	14	North West England; N = 985
**Hardcastle 2002** [24], 2001 [55], 2005 [56]	Qualitative^b^	ERS - Gym-and class-based activities	LC	10	East Sussex; N = 8; Age 43–77 years; all female
**Joyce 2010** [36]	Qualitative^c^	ERS (some GP referred) - Gym membership on prescription for patients with obesity related conditions	G	12	County Durham; N = 25 in community; GP ERS N = 5; F3 M2; 4/5 were >50 years old
**Khanam 2008** [45]	CSS^b^	GP referred ERS- 3 gym sessions per week (overweight women)	LC	Not stated	East London; N = 25; Age 30–60 mean age 47.3 (SD 9.1); all F; Bangladeshi; Muslim
**Lord 1995** [37]	Qualitative^c^	GP referred ERS −3 sessions per week	LC	10	Stockport; N = 252; Ag 30–55; F198 53 M53 1 Unknown; Socially deprived area; plus 6 referrers, 7 scheme providers
**Markland 2010** [46]	CSS^b^	GP referred ERS [no further description]	Not stated	10	UK (location not stated); N = 136; mean age 54.5; (SD 12.9); range 23–80; all F.
**Martin 1999** [25]	Qualitative^b^	GP referred ERS	LC/G	10	Margate Kent; N = 77; mean age 53 years; F39 M28
**Mills 2008** [26], 2012 [57]	Qualitative^b^	Primary care referred ERS including gym-based sessions and swimming	LC	26	Inner London; N = 17; mean age 54.7 (SD 12.4); range 31–68); F13 M4; plus 7 referrers, 4 scheme providers
**Morton 2008** [47]	CSS^b^	ERS (no detail) - Two sessions per week	LC	Ca 10–12	UK (location not stated); N = 30; mean age 51.9 years; F22 M8
**Murphy 2010** [27]	Qualitative^b^	GP referred ERS with discounted sessions in six centres	LC	16	Wales; N = 32 participants; CHD risk factors
Moore 2013 [58]					
**Myron 2009** [38]	Qualitative^c^	GP referred ERS - 2 centres	Varied	Not stated	UK (location not stated); N not stated. Mean age 42, range 20–72; 71 % F.
**Rahman 2011** [48]	CSS^b^	GP referred ERS; free of charge – 2 sessions per week	LC	12	UK (location not stated); N = 653; 18–83 years; F = 68.6 %. M = 31.4 %
**Schmidt 2008** [28]	Qualitative^b^	GP or health professional referred ERS -Specialist advice and low cost access to facilities	Not stated	20	Amsterdam, Netherlands; N = 523; Low SES and ethnic minority women aged 24–55
**Sharma 2012** [29]	Qualitative^b^	Health professional referred ERS - 2 supervised gym sessions per week	LC		South London; N = 9; 37–61 year; F4 M5; stroke survivors
**Shaw 2012** [30]	Qualitative^b^	GP referred for pre-exercise screening, health coaching (3 sessions) and community based exercise	Varied	52	Paisley, Scotland; N = 174; mean age 69.9 years; 43 M41; patients with stable coronary heart disease
**Singh 1997** [39]	Qualitative^c^	GP referred supervised ERS – 20 sessions free, 20 half price	LC	Not stated	South East London; N = 13; age range 30–61; F11 M2
**Stathi 2004** [31]	Qualitative^b^	Supervised ERS – gym and class based activities	LC	Not stated	South West England; N = 13; age range 63–79; F5 M8
**Tai 1999** [51]	Longitudinal^a^	GP referred ERS -Tailored programme of 20 sessions	LC	10	Inner London; N = 152; age range 16–75; F108 M44
**Taket 2006** [32]	Qualitative^b^	GP referred pilot ERS – three exercise consultations plus phone calls – walking, gardening, classes	Not stated	52	Inner London; N = 225; Age 44–65; F 22 M15; Type II diabetics; plus 14 non participants, 32 health professionals
Gauvin 2007 [59]					
**Taylor 1996** [40]	Qualitative^c^[within RCT]	GP referred ERS with 20 sessions at half cost – included rowing, cycling, step machine and treadmill sessions.	LC	10	Hailsham, East Sussex; N = 142; age range 40–70 years; patients with CHD risk factors
Taylor 1998 [60]					
**Walsh 2012** [41]	Qualitative^c^	Local authority subsidised exercise programme	Not stated	12	UK (location not stated); N = 2101, ≥age 45; chronic joint pain/osteo-arthritis; plus 88 scheme providers
**Ward 2007** [42]	Qualitative^c^	GP referred Welsh Heartlinks programme - ERS, Tai Chi, SlimSwim; motivational interviews	Varied	52	Merthyr Tydfil, Wales; N = 317; 24–88 years; F212 M105; plus 3 referrers
**Wiles 2008** [20]	Qualitative^a^	Physiotherapy referred ERS - 3 schemes	LC	Not stated	South England; N = 9; age range 18–78 years; 1 F 8 M; stroke survivors; plus 15 physios, 6 scheme providers
Wiles 2007 [61]					
**Wormold 2004** [33]	Qualitative^b^	GP referred ERS – 4 schemes	LC	10	North Yorkshire; N = 30; Age range 25–84; 20 F 10 M
**Wormold 2006** [34]	Qualitative^b^	Active Lifestyles service including ERS	Varied	10–12	Kingston upon Hull; N = 16; Mean age 53; range 15–73; 11 F 5 M; urban deprived;

Where more than one paper relates to a study, the main study paper is highlighted in bold

*CSS* Cross sectional survey, *ELC* Exercise and Life Style Centre, *ERS* Exercise referral scheme, *F* Female, *G* Gym, *LC* Leisure Centre, *M* Male, *SD* Standard deviation

Key to quality checklist scores [16]:

^a^All or most of the checklist criteria have been fulfilled, and where they have not been fulfilled the conclusions are very unlikely to alter

^b^Some of the checklist criteria have been fulfilled, and where they have not been fulfilled, or are not adequately described, the conclusions are unlikely to alter

^c^Few or no checklist criteria have been fulfilled and the conclusions are likely or very likely to alter

Most schemes (22 of 32) operated out of local authority leisure centres. Views expressed by participants focused primarily on gym-based and exercise class activities. The range of activities offered by providers was not always reported. Where reported (14 out of 22 local authority leisure centres) these included gym, exercise classes, swimming and walking.

### Findings from included studies

The included studies reported a wide range of participant views of factors affecting attendance at and successful completion of ERS and longer-term physical activity. We categorised the factors into 19 themes, ten of which may be described as extrinsic and nine as intrinsic to participants. Intrinsic factors define the participants, such as motivation and preferences. Extrinsic factors refer to a participant’s environment, such as family, scheme design or exercise setting.

### I. Extrinsic factors

#### Support

A number of themes highlighted the importance of support from providers, peers and family or friends for motivating scheme adherence and longer-term physical activity. Seventeen studies [20–28, 30, 31, 33–35, 40, 42, 44] identified good support and supervision from staff as a facilitator and its absence a barrier to adherence. Respondents often had concerns about exercising safely and also valued advice, support and encouragement from scheme providers [23, 24, 26–28, 30, 35]:

*‘I feel that if you were exercising and suddenly something happens, were they around? I didn't notice anyone (Participant). You were worried about harming yourself? (Researcher) Yes that's what it boiled down to’ (Beverly, aged 64)* [24].

Scheme members also felt that supervision was needed in order to build knowledge on how to use equipment, exercise effectively and improve fitness [22, 25, 27]. They commonly described how providers were needed to build or maintain their motivation to exercise [21, 24, 27, 33, 44]:

*‘It would be so easy to not bother when on your own’* [44].

Several studies highlighted the negative opinions expressed by scheme members about a perceived lack of provider support [20, 21, 35] and a positive feeling when adequate supervision was perceived [30, 34].

The desire for professional support beyond the end of the programme was a key concern for participants in eight studies [21, 23, 27, 28, 32, 33, 35, 36]. Its continuation beyond the programme was considered a facilitator [32, 33], and a lack of on-going support was seen as a barrier to continuing exercise [21, 23, 27, 28, 35, 36]:

*....most participants who dropped out of exercise post-completion of referral cited the removal of this Exercise Professional as the primary motivating factor* [35].

In addition to support from providers, scheme members greatly valued support from peers. Having a companion or buddy alongside was seen as a motivating factor by participants during the scheme [21–24, 29, 32, 35, 37, 39] and after [25]:

*‘It is nice because you have got a mixture of people you have got some people who are older than me and some who are younger than me, but we have that bit of a repartee between us, you know and we get on the bike and we say "we are off to high town now, come on all on your gears” So we make a laugh of it you see’* [23].

Engagement with others was seen as an aid to integration and enjoyment of exercise referral schemes in 15 studies [21–24, 26–30, 33, 35–37, 44, 45]:

*Some said they found it encouraging that the group was made up of friendly participants with similar health conditions, and this is also mentioned as a stimulus for continuing to exercise: 'If she can do it, maybe I can too’* [28].

*'gym is a lonely place'* [26].

Participants also benefited from group activities in the company of like-minded companions rather than solitary exercise, as reported in six studies [20, 21, 25, 26, 30, 33]:

Beyond support from providers and peers within exercise referral schemes, participants found external support from family members and friends, particularly from a spouse, encouraged them to participate in physical activity [23–25, 40]. A lack of support was found to discourage uptake and adherence [40, 45], as reported in six studies exploring the theme of external support.

#### Scheme setting and accessibility

Many studies discussed participant views on scheme settings (gym or leisure centre environment) and accessibility (location, travel and cost). Respondents in 9 studies described feeling uncomfortable and intimidated in the unfamiliar gym environment [21, 22, 24–27, 33, 35, 40]. This may be related to a perceived image of other users being fit, slim, young and beautiful [22, 24, 25, 33, 35] together with participants’ own low self-esteem and body image [21–24, 28, 29, 40]:

*‘I felt very uncomfortable every time I entered the gym to the extent I felt like a freak (F/38/460)’* [21]*.*

*Alison ‘I thought it was probably going to be all, you know, young and beautiful who were all frightfully good at everything’ (2 fg1 122–3). Claire ‘I didn’t know what to expect you know, but I have felt a bit like you that it might be all beautiful young things in their leotards and what not’* [22].

Participants also reported concerns about using gym equipment [21, 22, 35]:

*‘The technology totally overwhelmed me’ (Participant 0201). ‘I ruined one machine; I’m just not inclined that way’ (Participant 0205)* [35].

Negative opinions about the noise, volume or type of music played were expressed in six studies [22, 25, 26, 35, 40, 45]. Televisions content was perceived to be inappropriate [45], not to personal taste [35], or too loud/quiet [22]. Conversely respondents in three studies [22, 24, 35] found music or television helpful in distracting them from feelings of anxiety in an unfamiliar environment or alleviating boredom. The quality of the facilities as a deterrent to attendance by participants was reported in four studies [22, 30, 35, 40], although one [22] reported mixed views on whether this was a deterrent.

Besides scheme setting, the included papers also covered aspects of accessibility that presented barriers to participant adherence: their location (distance to travel) [21, 27, 28, 32, 35, 45] and the perceived safety of the location [19, 26, 28], difficulties reaching the activities by public transport [19, 25, 27, 32, 34, 45], and their cost [21, 26, 28, 30, 35, 51].

#### Timing and content of sessions

Two themes related to participant views on scheme administration (scheduling and variety of activities). Inconvenient timing of sessions was viewed by respondents as a barrier to attendance [21, 24, 26, 27, 30, 32, 35, 37, 40, 47] mainly in relation to clashes with work hours or childcare commitments:

*‘…I need to be able to fit it around my work … they need to provide times at the weekends or in the evening.’ (Female, Black, 45–50)* [32].

Activities scheduled during off-peak gym times allowed attendance at times when the environment was ‘less intimidating’ [40]. However, this was inconvenient for day-time workers [40]. Within the broader scheduling theme, respondents in three studies [30, 35, 40] described ‘rigid’ appointment times or lack of flexibility in scheduling as a barrier to attending.

Participants’ views on the range of activities offered by schemes and their preference for various exercise types were reported in 12 papers [21, 22, 24, 26, 27, 30–33, 35, 45, 49]. Views on gym based activities varied with some reporting a liking for a safe environment unaffected by the weather [24, 31, 33], whilst others disliked gym exercise, citing boredom [21, 24, 35], preference to be outside [21] or a dislike of lifting weights [24]:

*A number of participants referred to exercise sessions as being `boring', often citing the monotony of the programme, or the machines as the root cause* [35].

While many valued the range of existing activities, others wanted more variety [27, 30, 31, 33]. Preferences for other forms of exercise were discussed, including group-based activities such as dance, aerobics or yoga [22, 24, 32, 33, 45], swimming [21, 22, 27, 33, 45] or outdoor activities such as walking [21, 22, 45] and cycling [21, 22].

### II. Intrinsic factors

#### Individualisation

Two themes related to individual preferences, religion or culture affecting adherence to a scheme. Eight studies exploring the theme of personalised service described scheme members wanting individualised attention and an exercise schedule tailored to their needs, ability or preferences [21, 24–27, 32, 33, 35]. For example,

*‘I don't like particularly just being a number I like the fact that someone was paying attention to me’ (Yvonne, aged 65, at week five of the programme)* [24].

*‘They were interested in dovetailing it to me personally…feel healthier as a result.’ (Male, White, 51–65)* [32].

The views of participants from minority religions and cultures also highlighted the need for individualisation of schemes. Scheme members [19, 28, 45] clearly identified the need for women-only sessions to meet the religious needs of Muslim women:

*The health and fitness adviser was also aware of possible religious barriers, specifically the need for Muslim women to exercise in a men-free environment, thus respecting male–female dynamics within Islam. In addition, it was important not to hold women only sessions on Fridays (Jumma), the Muslim holy day* [19].

Language problems and an inability to communicate effectively were identified as barriers to uptake and adherence by participants in two studies [19, 45].

*‘I turned back at the door because I knew I wouldn’t be able to understand what the lady at the desk would say’* [19].

#### Goals and motivation

Studies exploring scheme members’ goals and motivations reported a range of views from which few clear themes emerged. However, a range of perceived improvements in physical health and mental well-being were reported.

Despite the nature of the intervention, increased physical activity was not the main goal for participants when joining a scheme. More common motivations were improved health, reducing existing health problems or avoidance of ill health, as reported in seven [20–24, 27, 39] of the nine studies [20–24, 27, 33, 39, 45] discussing goals. For example,

*‘I don't want to be sitting in a wheelchair do I in another ten years. I just want to be active and keep going’* [23].

Participants tended to focus on having better fitness levels [21, 23, 24, 33], or aimed to lose weight [21, 22, 24, 33]. Social inclusion goals, such as ‘getting out of the house’ or ‘making friends’ were reported in three studies [21, 22, 24].

Motivation was explored in 17 studies [19, 21–28, 32, 34–36, 40, 45, 47, 48] and varied without clear themes emerging other than that participants felt they should exercise [24, 26, 28] and lacked self-motivation [21, 24–26, 32, 48]. Lack of time as a result of personal commitments was identified as a barrier in all four studies [21, 24, 32, 40] exploring the theme. Personal commitments to work, family or social demands made it difficult to find time to exercise. Whether enjoyment of exercise was perceived by participants as a crucial factor for joining or completing ERS programmes is unclear: studies [21, 22, 24, 26, 35] exploring this theme reported that some participants enjoyed the activity itself [21, 22, 24, 26, 35] whereas others, whilst not enjoying the activity, appreciated the associated benefits such as satisfaction in maintaining willpower to achieve their goals [22, 24] or the physical benefits [22, 24, 26, 35]. Eleven studies [21, 23–26, 31, 32, 34, 40, 41, 47] explored health concerns, which were reported as a facilitator for those desiring health improvement [21, 24, 26] but a barrier for those with concerns of injury or exacerbation of a condition [21, 23–26, 32, 41, 47].

Participants in twenty-one studies [21–27, 29, 31–37, 39, 40, 42–44, 50] described outcomes resulting from participating in an ERS. A range of improvements in physical health and mental well-being were reported. The most common improvements were to aspects of physical health (general physical fitness [21–24, 26, 27, 29, 31–35, 40, 42], general health benefits [21–24, 31–34, 36, 37, 39, 40, 42, 44, 50], weight loss or improved tone [21, 23, 24, 26, 27, 32, 34, 42, 50] and increased physical activity [24, 31–35, 40, 43, 50]). Notably, improvements in mental well-being were reported in 14 studies [21–24, 26, 27, 29, 31, 33, 34, 36, 40, 42, 50].

*… ‘I feel totally at one, totally alive and totally happy’ [Mary, 1i3 73]* [22].

Respondents also described improved social engagement [22, 24, 31, 33, 34, 40, 42, 50] and an increase in personal autonomy [21, 22, 24, 26, 27, 29, 31, 33, 34].

*When recalling ERS participation, interviewees expressed the importance of their own personal qualities to successful recovery and increasing independence, attributing improvements to internal factors such as motivation, willpower and self-determination* [29]

Five studies [21, 27, 31, 33, 37] noted a perceived poor or negative outcome of ERS. Participants reported negative effects on general health and mental health [21], an exacerbation of specific health problems [37], a disappointment over failure to lose weight [27] and the view that not all could benefit from increased social engagement. Lack of benefit from social engagement was an issue for those with caring commitments or because a gym setting was found less conducive to engagement [31, 33].

There was limited information available on participant views of on-going exercise after completion of exercise referral schemes. These were explored in five studies [23, 24, 27, 34, 35]. Establishing regular exercise routines and exercise becoming a habit were perceived by participants as facilitators of long-term physical activity [24, 27, 34].

*'I've started walking to the shops, where I took the car in the past*’ [34].

Similarly, the risks of falling out of the habit of exercise [23, 24] and loss of social support when scheduled exercise sessions with similar individuals finished [23, 27, 35] were identified as barriers. For example,

*…others expressed concerns that they might struggle to maintain motivation without a commitment to exercise in a set time and place and the loss of social support* [27].

### III. Framework for successful implementation of exercise referral schemes

We summarized the participant views according to how they might be taken into consideration when implementing an exercise referral scheme (Table 2). To achieve this we mapped the themes against the core concepts of the PARiHS (Promoting Action on Research Implementation in Health Services) Framework [52], a conceptual implementation framework based on evidence on the critical success factors to successful implementation of interventions in practice. Within the Framework, successful implementation of an intervention is associated with the evidence supporting its use, the context in which it is being introduced, and the way in which it is facilitated to achieve successful outcomes. The barriers and facilitators are mapped against these core concepts on a high (facilitator) to low (barrier) continuum. This framework has been adapted to map out the *identified* barriers and facilitators to implementation of exercise referral schemes within the same three dimensions, envisaged from the perspective of ERS participants.

**Table 2:** PARiHS Framework: Critical success factors to maximise adherence to Exercise Referral Schemes by participants

Dimensions	LOW implementation (Barriers)	HIGH implementation (Facilitators)
**Context** Socioecological context of ERS patients (eg personal characteristics, home, work and family)	**Concerns about worsening health problems** was a barrier to adherence for some participants **Lack of time** as a result of personal commitments to work, family, role as a carer or social demands **Loss of social support** after the intervention **Lack of external support** from family members, particularly a spouse **Not accommodating cultural/religious requirements :** eg, language problem and the inability to communicate effectively with providers	**External support from family members** particularly a spouse **Cultural/religious sensitivity** such as women-only activities and consideration of religious holy days **Maintaining routine:** Making exercise a habit was viewed as important to ongoing physical activity beyond the ERS scheme
**Evidence** Could include research evidence, clinical experience, patient experience and local data	***Participant experience*** **Perceived poor/negative outcomes of ERS** included general and mental health, exacerbation of specific health problems, disappointment over failure to lose weight and not benefitting from increased social engagement **Poor perceptions of the intervention atmosphere and environment:** Feeling uncomfortable in an ‘intimidating gym environment‘; Dislike of music/tvs in gyms; Difficulties operating gym equipment; Poor quality facilities **Dislike of gym-based exercise** due to boredom, preference for being outside	***Participant experience*** **Perceived improvements:** Physical health improvements were the most commonly described; Others included weight-loss and physical activity, mental wellbeing and personal autonomy, social engagement - both during and after the programme **Liking for gym-based activities** because of its safe environment and weather independence **Desire for range of different types of physical activities** including dance, aerobics, yoga, swimming, or outdoor activities such as walking and cycling **Group activities** valued, with participants liking being in the company of like-minded companions rather than solitary exercise
**Facilitation** Factors related to the presence or absence of how the ERS scheme facilitates participation and progress	**Perceived lack of sufficient support and supervision from providers** **High cost** of exercise facilities, particularly after a subsidised ERS scheme **Inconvenient scheduling** eg activity timings clashing with work hours or child care **Lack of ongoing professional support** after the ERS **Venue Location Problems:** Long distance to travel, difficulties with public transport, perceptions of venue locations not being safe for women	**Support and supervision from providers** to help guide safe and efficient exercise, provide equipment, knowledge and motivation **Peer support** highly valued, specifically in relation to (i) having a companion/buddy to do the activity with during the scheme; (ii) engagement with others aiding integration and enjoyment **Individualised and personalised service** including an exercise programme tailored to user needs, ability, health status, preferences, goals and values **Off-peak scheduling:** The gym environmental was perceived to be less intimidating during off-peak hours. However, this was inconvenient for day-time workers **Continuing professional support after the ERS programme** was desired and described as a facilitator

The bold highlights the identified themes

## Discussion

The NICE guidance on exercise referral schemes (PH54) [9] recommends incorporating core behaviour change techniques outlined in a separate guideline, NICE public health guidance 49 (PH49) [13]. This provides advice on how such techniques can be applied to interventions aimed at changing damaging behaviours. The themes identified here give providers a perspective on what makes it more or less likely that scheme participants will make sustained behaviour changes.

One recommendation is that interventions advise on and arrange social support. The views presented in this review show that support from providers, peers, family and friends is a strong facilitator for adherence. Another recommendation is that interventions recognise when people are open to change. Identified themes that focus on goals, motivation, enjoyment of exercise, existing health concerns and personal commitments provide insight into how participants feel about incorporating exercise into their daily lives. Preferences on scheme setting and accessibility (cost, location, travel, setting) and the timing and content of activities (scheduling of activities and types and variety of activity) are valuable background information for agreeing goals and developing action plans to help change behaviour, another core behaviour change technique recommended. The exercise referral guidance [9] also recommends that schemes tailor interventions to individual needs. Identified themes relating to religion and culture and individualised, personalised service are factors that influence adherence and are worth taking into account. Participant views on making exercise a habit post-programme could help to develop coping plans to prevent relapse.

The included studies in this systematic review report a large number of participant views on multiple facets of ERS adherence. Unfortunately no common themes emerged for participant motivation, likely to be a key element in promoting of physical activity. This may be due to the heterogeneity across studies, an issue pointed out by Pavey and colleagues in a systematic review of levels and predictors of exercise referral schemes [53].

### Limitations

Although the quality of studies overall was judged as moderate, a number of qualitative studies were generally well conducted research within PhD theses. The nature of the qualification requires a single investigator. This meant the studies were assessed as moderate, despite being otherwise well conducted. Other studies assessed as low were process evaluations that were not designed with formal methodologies. Nevertheless they were of value in corroborating data from other studies.

The available evidence was limited for some populations: ethnic minorities, people with disabilities and low socio-economic groups. The studies generally reported on older age groups and included more women than men. Most schemes included a range of activities. However, views expressed by participants focused primarily on the gym and exercise classes.

## Conclusion

This paper describes the views of a wide range of scheme participants identified in a systematic review of barriers and facilitators of adherence to exercise referral schemes [14] informing NICE’s 2014 public health guidance on this topic [9]. These findings (summarised in Table 3) were consistent across the research and provide valuable insights that commissioners and providers should consider to maximise the chances of adherence to a scheme and successful outcomes for participants. Further research should concentrate on schemes that have been adapted to reflect these findings.

**Table 3:** Summary of themes

Theme	Facilitator	Barrier
Support		
Professional advice and supervision (during and after ERS)	√	
Encouragement and support from peers and family or friends	√	
Social engagement with other participants	√	
Setting/accessibility		
Accessible location	√	
Good public transport links	√	
Loud music/TV in gym	√	√
Gym environment		√
Complex gym equipment		√
Poor quality facilities		√
Cost		√
Timing and content		
Variety of exercise options	√	
Flexible session times	√	
Individualisation		
Tailored exercise programmes	√	
Lack of cultural awareness and language difficulties		√
Goals and motivation		
Perceived benefits in physical and mental health	√	
